# Macrovascular Function in People with HIV After Recent SARS-CoV-2 Infection

**DOI:** 10.3390/jvd4010004

**Published:** 2025-01-26

**Authors:** Ana S. Salazar, Louis Vincent, Bertrand Ebner, Nicholas Fonseca Nogueira, Leah Krauss, Madison S. Meyer, Jelani Grant, Natalie Aguilar, Mollie S. Pester, Meela Parker, Alex Gonzalez, Armando Mendez, Adam Carrico, Barry E. Hurwitz, Maria L. Alcaide, Claudia Martinez

**Affiliations:** 1 Division of Infectious Diseases, Department of Medicine, University of Miami Miller School of Medicine, Miami, FL 33136, USA; 2 Department of Internal Medicine, University of Miami/Jackson Memorial Hospital, Miami, FL 33101, USA; 3 Division of Cardiovascular Medicine, Department of Medicine, University of Miami Miller School of Medicine, Miami, FL 33136, USA; 4 Department of Medicine, Wayne State University School of Medicine, Detroit, MI 48201, USA; 5 Division of Cardiology, Department of Medicine, Johns Hopkins University, Baltimore, MD 21218, USA; 6 Behavioral Medicine Research Center, University of Miami Miller School of Medicine, Miami, FL 33136, USA; 7 Department of Psychology, University of Miami Miller School of Medicine, Miami, FL 33136, USA; 8 Division of Endocrinology, Diabetes and Metabolism, University of Miami Miller School of Medicine, Miami, FL 33136, USA; 9 Department of Public Health, University of Miami Miller School of Medicine, Miami, FL 33136, USA

**Keywords:** HIV, SARS-CoV-2, flow-mediated dilation, arterial stiffness, macrovascular function

## Abstract

**Background:**

People with HIV (PWH) are at increased risk of vascular dysfunction and cardiovascular disease (CVD). SARS-CoV-2 infection has been associated with acute CVD complications. The aim of the study was to as-sess macrovascular function as an early indicator of CVD risk in PWH after mild SARS-CoV-2 infection.

**Methods:**

PWH aged 20–60 years, with undetectable viral load (RNA < 20 copies/mL), on stable antiretroviral therapy (≥6 months) and history of mild COVID-19 (≥30 days) without any CVD manifestations prior to enrollment were recruited. Participants were excluded if they had history of diabetes mellitus, end-stage renal disease, heart or respiratory disease. Participants were matched 1:1 to pre-pandemic PWH. A health survey, surrogate measures of CVD risk, and macrovascular function (brachial artery flow-mediated vasodilation and arterial stiffness assessments via applanation tonometry) were compared between group.

**Results:**

A total of 17 PWH and history of COVID-19 (PWH/COV+) were matched with 17 PWH without COVID-19 (PWH/COV−) pre-pandemic. Mean age (45.5 years), sex (76.5% male), body mass index (27.3), and duration of HIV infection (12.2 years) were not different between groups. Both groups had comparable CVD risk factors (total cholesterol, LDL, HDL, systolic and diastolic blood pressure). There were no differences in measures of flow mediated arterial dilatation or arterial stiffness after 30 days of SARS-CoV-2 infection.

**Conclusions:**

After recent SARS-CoV-2 infection, PWH did not demonstrate evidence of macrovascular dysfunction and increased CVD risk. Results suggest that CVD risk may not be increased in people with well-controlled HIV who did not manifest CVD complications SARS-CoV-2 infection.

## 1. Introduction

Severe acute respiratory syndrome coronavirus 2 (SARS-CoV-2) has infected over 101 million people and accounts for more than 1 million deaths in the United States [1]. Growing evidence of extra-pulmonary complications from COVID-19 has demonstrated associations with myocarditis, acute myocardial injury, and other cardiovascular (CV) complications [2,3]. In part, these complications are believed to be mediated by the binding of the viral spike protein on the surface of SARS-CoV-2 to the human angiotensin-converting enzyme-2 receptor. This receptor is highly expressed in the heart, lungs, vascular endothelium, and via the renin–angiotensin–aldosterone system [4]. SARS-CoV-2 infection may also lead to elevated levels of inflammatory cytokines, such as IL-6, IL-7, and IL-22, among others, which may increase oxygen radical formation and damage CV cells [5]. Furthermore, it is possible that activated T-cells and macrophages may infiltrate the infected myocardium and vascular endothelium, respectively, resulting in macrovascular dysfunction. This inflammatory process is characterized by vascular endothelial dysfunction and arterial stiffness, which are well-established precursors of CV disease [6].

As new studies create a better understanding of the systemic impact of SARS-CoV-2 infection, it is critical to explore the overlapping effects of established CV risk in people living with HIV (PWH). Both SARS-CoV-2 and HIV damage the vascular endothelium via chronic immune activation, cytokine elevation, such as IL-6 and TNF-α, and direct vascular injury via viral interaction with endothelial cells [5,7]. This common pathophysiology pathway increases CV risk in PWH, especially following SARS-CoV-2 infection.

Emerging research highlights the importance of understanding how comorbidity in vulnerable populations such as PWH influence the progression of CV disease [8]. Aside from the immediate physiological consequences of HIV combined with COVID-19, lifestyle, access to care, and treatment compliance also influence CV outcomes [9]. For instance, disparities in access to and quality of healthcare during the COVID-19 pandemic caused ART interruptions and led to increased immune dysregulation and CV risk [10,11]. Moreover, the added burden of chronic disease management in the midst of a global epidemic stresses the importance of combining CV care with everyday HIV care [12]. This increased awareness of systemic healthcare inequity underscores the need to not only understand the biological aspect of HIV, but the structural inequity that influences CV health in PWH post-COVID-19, as well [13].

There is evidence to support a link between COVID-19, HIV, and CV complications [14]. People with both HIV and SARS-CoV-2 are at risk of developing CV complications and immune dysregulation [15]. An upsurge in pro-inflammatory cytokines, vascular endothelial dysfunction, and COVID-19-induced arterial stiffness are also significant pathophysiological features of HIV [7]. PWH with low CD4 or untreated HIV are at a higher risk for reduced health outcomes resulting from COVID-19 infection [16]. However, CV risk is comparable in PWH with a reduced viral load effectively controlled by antiretroviral therapy to those without HIV [17]. These results illustrate the need for tailored and optimized HIV treatment to avoid overlapping risks.

COVID-19 infection also causes an increase in vascular endothelial function in at-risk groups like PWH [18]. SARS-CoV-2 causes vascular endothelial damage by binding ACE2 receptors and activating the immune system [19]. This results in an increase in CV events like myocarditis, arrhythmia, and thrombosis [20]. PWH often experience chronic immune activation and vascular inflammation, even when their viral load is controlled with regular antiretroviral therapy [21]. This is likely to produce a synergistic effect that elevates PWH’s risk of developing immediate and long-term CV complications [22]. However, certain antiretrovirals have been found to be cardioprotective, such as integrase inhibitors, especially in the context of HIV treatment [23,24]. However, these overlapping mechanisms highlight how important it is to study microvascular function in PWH following COVID-19 infection.

Based on the immunosuppressive nature of HIV, PWH are thought to be at an increased risk of COVID-19 infection [25–27]. Large cohort studies suggest that PWH who have a low CD4+ T-cell count and are not on antiretroviral medication may be at increased risk of COVID-19-associated mortality [28–30]. However, emerging studies also suggest this immunosuppressive nature and low CD4 count may protect PWH from developing the “cytokine storm” seen during COVID-19 infection [27,31].

While cardiovascular problems in the general population and in people who have severe COVID-19 are well-documented, the cardiovascular consequences of mild SARS-CoV-2 infection in PWH have been poorly studied. It is important to note research has focused primarily on individuals living with highly advanced or uncontrolled HIV, with a lack of research on those with well-controlled HIV, who make up a growing share of PWH. Moreover, there is no literature that highlights macrovascular function as a CV disease risk factor in PWH.

This study bridges these inconsistencies using advanced diagnostic tools such as flow-mediated dilation (FMD) to assess macrovascular function and arterial stiffness in PWH [32]. These findings bring novel insights into subclinical cardiovascular disease by examining individuals living with well-controlled HIV as well as SARS-CoV-2 and can help inform future preventive practice toward this disadvantaged patient population.

## 2. Methods

### 2.1. Participants

This cross-sectional study included participants enrolled at the Clinical Infectious Diseases Research Unit at the University of Miami’s Miller School of Medicine, in collaboration with the Miami Center for Aids Research (CFAR), between November 2017 and March 2021. Recruitment methods have been previously described elsewhere [33]. In short, participants were enrolled if they were 20–60 years old, had a documented HIV-1 diagnosis, an undetectable viral load (RNA < 20 copies/mL), were on stable antiretroviral therapy (≥6 months), and with a history of COVID-19 infection (≥30 days) confirmed by nucleic acid amplification testing RT-PCR. All participants had mild COVID-19 symptoms without hospitalization. Participants with comorbidities, such as diabetes mellitus, end-stage renal disease, heart disease, and respiratory disease, were excluded to ensure vascular endothelial outcomes were not confounded by pre-existing conditions that independently affect CV risk and worsen vascular endothelial function. This approach was essential to isolate the impact of HIV and SARS-CoV-2 infection on microvascular function. PWH and COVID-19 (PWH/COV+) were matched 1:1 based on age, sex, ethnicity, race, and BMI to historical controls from our studies prior to the COVID-19 pandemic (PWH/COV−) [34].

### 2.2. Ethical Approval

This study was approved by the University of Miami’s Institutional Review Board (20200748) on 23 July 2020 and all participants provided their informed consent prior to assessment. Research on human subjects was conducted in conformity with the University of Miami’s ethical standards and the Helsinki Declaration of 1975, revised in 2013.

### 2.3. Study Procedures

A questionnaire was administered in-person, by telephone, or online, based on participants’ preference, using REDCap electronic data capture tools hosted at the University of Miami. REDCap (Research Electronic Data Capture) is a secure, web-based software platform designed to support data capture for research studies, providing (1) an intuitive interface for validated data capture; (2) audit trails for tracking data manipulation and export procedures; (3) automated export procedures for seamless data downloads to common statistical packages; and (4) procedures for data integration and interoperability with external sources [35]. Participants had the option to respond to the survey in English or in Spanish. Demographic data, comorbidity, HIV history, concomitant medication, tobacco use, substance use, and prior symptoms of COVID-19 (≥30 days but <1 year) were collected. Subsequently, participants were invited to an in-person visit to collect anthropometric and vascular measurements and biological samples.

Anthropometric measurements (height, weight, and waist circumference) and vital signs (heart rate and systolic and diastolic blood pressure) were collected prior to vascular assessment. Body mass index (BMI) and mean arterial pressure (MAP) were calculated accordingly. The macrovascular assessment included the following elements: B-mode ultrasound brachial artery reactive hyperemia testing, allowing for the measurement of endothelial-dependent FMD, and applanation tonometry, to obtain measurements of central (aortic) and peripheral (radial and femoral) arterial stiffness [36]. Blood samples were collected following, at minimum, an 8-h fast. Biological samples were analyzed for CMP, CRP, insulin, and a lipid panel by the Diabetic Research Institute at the University of Miami.

### 2.4. Fasting Glucose, Insulin, and Lipid Profile

A venous sample totaling 5 mL was obtained and assays performed to obtain fasting plasma glucose (FPG) and insulin (FPI) levels. The homeostasis model was used to derive an insulin resistance estimate (HOMA-IR) [37]. In addition, total cholesterol (TC), triglycerides (TGs), HDL-cholesterol, and high-sensitivity C-reactive proteins (CRPs) were measured on a Roche 6000 Auto-Analyzer (Roche Diagnostics, Basel, Switzerland). Automated chemistry and immunoassays exhibit interassay coefficients of variation consistently less than 5%. LDL-cholesterol was calculated by the Friedewald equation [38].

### 2.5. Statistical Analysis

Group comparisons (PWH/COV+ and PWH/COV−) were examined using the mean/median for continuous variables, and frequencies for categorical variables. Descriptive data were analyzed using a chi-square and *t*-tests for parametric and non-parametric variables, respectively, where appropriate. Significance was established at α = 0.05. Statistical analyses were performed using RStudio statistical software (version 4.0.3; R Foundation for Statistical Computing, Vienna, Austria) and SAS 9.4.

## 3. Results

### 3.1. Participant Characteristics

This study compared 17 PWH/COV+ to 17 PWH/COV−. The sociodemographic and clinical characteristics of the studied groups are described in Table 1.

### 3.2. Surrogate Measures of CVD Risk and Macrovascular Function

Table 2 displays differences in measures of CVD risk between the groups. Both groups had comparable systolic and diastolic blood pressures and resting heart rates. There were no statistically significant differences between the groups regarding central adiposity, indexed by waist circumference, insulin resistance, assessed by HOMA-IR, nor regarding fasting plasma insulin or fasting plasma glucose levels. Similarly, levels of CRP, TGs or HDL, TC, LDL, TC/HDL or TG/HDL were comparable between both groups. There was a trend in the PWH/COV+ group toward lower FMD and higher central augmentation indices, but there were no significant differences in the measurement of macrovascular function in either group of PWH.

## 4. Discussion

This study represents the first cross-sectional study assessing measures of CVD risk, including macrovascular function, among PWH who had a recent COVID-19 infection but no CVD complications. We found no significant difference in CVD risk factors, nor in measures of macrovascular function among PWH with and without a SARS-CoV-2 infection.

Therefore, it is possible that no acute CVD complications at the time of COVID-19 infection could mean there is no increased risk of CVD in PWH. This is of particular relevance to the HIV population as HIV infection is an independent factor that increases the risk of CVD. This is, in part, due to the impact of chronic inflammation of viremia on macrovascular function and to the status of immune activation in the context of well-controlled HIV infection [39]. Of note, participants in this study all had undetectable VLs and were on stable ART therapy. However, larger and longer follow-up studies are needed to further define CVD risk after COVID-19 among PWH.

Previous studies comparing vascular endothelial dysfunction in people living without HIV with and without SARS-CoV-2 have demonstrated that post-COVID-19 patients had increased arterial stiffness, intima–media thickness (IMT), and von Willebrand factors compared to healthy controls [39,40]. However, it is possible that the degree of vascular endothelial dysfunction is associated with the severity of COVID-19 infection. In a small cross-sectional study of patients hospitalized with COVID-19 in Italy, patients who developed pneumonia, respiratory distress, or hypoxia had lower brachial flow-mediated dilation (FMD), where a lower FMD was associated with increased risk of ICU admission or in-hospital death [41]. In contrast, individuals in our study’s population had only mild SARS-CoV-2 symptoms, such as no hospitalization, pneumonia, or severe respiratory complications. These findings may help explain why those within our cohort lacked significant vascular endothelial dysfunction markers, which reflect a milder course of disease in this population. This study highlights critical gaps in the literature, specifically long-term CV effects in PWH following infection with COVID-19.

Many recent studies have looked at endothelial dysfunction as a significant component of COVID-19 disease later in life [42]. Long-term vascular endothelial damage was associated with chronic vasoconstriction, arterial stiffness and reduced FMD following COVID-19 infection across populations of different ages [43,44]. For example, analyses of endothelial function in COVID-19 patients have shown elevated von Willebrand factors and endothelial activation [45]. These all suggest that there are enduring vascular effects after mild infection. They are particularly relevant for PWH, who are already at an elevated cardiovascular risk due to long-term immune activation and inflammation [39].

In order to properly address long-term CV implications for PWH recovering from COVID-19, longitudinal studies are required. Future investigations should focus on whether persistent inflammation or subclinical vascular endothelial changes increase the risk of developing acute or chronic CV disease over time. They should also study how HIV therapy affects CV function, as certain ARTs protect or damage CV function. This future research should focus on the mechanism underlying the influence of ART regiments on vascular endothelial dysfunction, arterial stiffness, and cardiovascular risk. Understanding this relationship is important given that certain regimens such as Tenofovir have been found to be cardioprotective while other regimens cause CV damage [46]. This future research can guide personalized treatment options to optimize CV heath in PWH.

As there are ever-changing SARS-CoV-2 variants and vaccines, future individualized health plans deserve longitudinal research. In addition, imaging and biomarkers may allow for the early detection and prevention of CV disease in PWH using advanced diagnostics. Future studies must also look at the extent to which health factors, including care and income, compound these risks. It is necessary to develop personalized interventions for these compounded risks if healthcare providers are to ensure equitable access to and treatment for PWH. Individual and systemic engagement is critical to promote health in this at-risk population.

There are some limitations worth noting in this study. This was a pilot study conducted early in the COVID-19 pandemic, making in-person recruitment/enrollment a factor that limited our ability to obtain a large sample size. Through the exclusion of participants with specific comorbidities, the generalizability of the findings was limited to PWH without coexisting conditions. The study size may be inadequate to detect important group differences and may increase the probability of type II errors. Moreover, unmeasured confounding factors, including diet, physical activity, and genetics, may have affected the study’s findings by masking low-level differences in macrovascular function between the groups. In addition, biological differences in endothelial function and arterial stiffness were uncontrollable, as both depend on stress and environmental exposure. Such unmeasured confounding factors underscore the need for larger, better-controlled studies to explain this group–to–group variation. It is plausible that deleterious effects resulting from infection may become evident with a greater follow-up time, or that the risk of developing CVD may be accelerated over time, if not sub-acutely increased. Results may not be applicable to the current state of the pandemic, characterized by novel viral variants and overall decreased severity and CV outcomes, nor to PWH not on antiretroviral medication. Nonetheless, this study represents an important first step in understanding the short-term risks of macrovascular dysfunction among PWH with a history of COVID-19.

## 5. Conclusions

Following a recent SARS-CoV-2 infection, PWH did not demonstrate evidence of macrovascular dysfunction nor increased CVD risk. Results suggest that CVD risk may not necessarily be increased in people with well-controlled HIV who do not manifest CVD complications resulting from SARS-CoV-2 infection. However, additional longitudinal studies are required to evaluate long-term cardiovascular outcomes and explore subclinical progression with complete surveillance and treatment for this vulnerable population.

## Figures and Tables

**Table 1.** Sociodemographic, clinical, and HIV-related characteristics of participants by study group.

	HIV + COVID+ (n = 17)	HIV + COVID− (n = 17)	Comparison ^a^

Measures	Mean SE	Mean SE	

**Demographics**			

Age (yrs)	47.1 ± 2.5	44.0 ± 1.6	0.30
Sex (% men)	76.5	76.5	1.00
Ethnicity/Race (%)			0.29
Black	17.6	23.5	
Hispanic	82.4	64.7	
Non-Hispanic white	0.0	11.8	

**Clinical Characteristics**			

BMI (kg/m^2^)	26.5 ± 1.1	28.1 ± 0.8	0.40
BMI: overweight (%)	58.8	58.8	1.00
obese(%)	29.4	29.4	1.00
Dependence score (U)	17.0 ± 4.1	18.5 ± 3.7	0.78
Abuse score (U)	13.0 ± 2.5	15.8 ± 3.1	0.48

**HIV-related Characteristics**			

Known HIV duration (yrs)	13.0 ± 1.6	11.0 ± 2.5	0.51
Undetected viral load (%)	100.0	100.0	1.00

Abbreviations: BMI = body mass index

a χ^2^ or ANOVA group differences.

Annotations: The PWH COVID+ group non-significantly reflected differences in metabolic health post-infection. Disparities in COVID-19 exposure and outcomes based on population are reflected by an increased percentage of Hispanic-identifying participants in the PWH COVID+ group. This was a well-matched group; there was a similar rate of HIV duration and viral suppression between the groups, reflecting differences in vascular measures that are not likely to be influenced by HIV management.

**Table 2.** Comparison of subclinical cardiometabolic measures between HIV+ studied groups.

	HIV + COVID+ (n = 17)	HIV + COVID− (n = 17)	Comparison ^a^

Measures	Mean SE	Mean SE	

**Subclinical CVD risk**			

Waist circumference (cm)	97.2 ± 3.4	96.1 ± 2.4	0.79
HOMA-IR^c^ (mass units)	3.2 ± 0.7	4.1 ± 0.9	0.41
FPG ^c^ (mg/dL)	96.0 ± 2.4	96.3 ± 2.2	0.94
FPI^c^ (μU/mL)	13.4 ± 3.2	12.5 ± 2.0	0.49
CRP (mg/L)	3.0 ± 0.6	1.9 ± 0.5	0.11
TGs ^c^ (mg/dL)	156.4 ± 28.5	173.7 ± 34.0	0.70
TC (mg/dL)	203.4 ± 116	198.7 ± 9.7	0.76
LDL (mg/dL)	129.1 ± 10.0	118.1 ± 7.1	0.37
HDL (mg/dL)	47.5 ± 2.3	45.9 ± 3.3	0.96

**Subclinical CVD risk**			

TC/HDL	4.5 ± 0.2	4.7 ± 0.5	0.72
SBP (mm Hg)	124.0 ± 3.1	119.7 ± 4.1	0.40
MAP (mm Hg)	92.3 ± 2.3	89.9 ± 2.4	0.47
DBP (mm Hg)	74.5 ± 2.0	75.0 ± 1.8	0.86
Heart rate (bpm)	63.2 ± 2.2	66.4 ± 2.3	0.34

**Vascular Function**			

FMD (%)	6.3 ± 0.7	7.4 ± 0.8	0.34
cAIx (%)	21.7 ± 3.2	16.4 ± 3.6	0.28
cfPWV (m/s)	7.3± 0.5	6.7 ± 0.3	0.26
crPWV (m/s)	8.5 ± 0.8	8.5 ± 0.4	0.98
cPP (mm Hg)	34.4 ± 1.7	30.2 ± 1.5	0.08

Abbreviations: BMI = body mass index; HOMA-IR = homeostatic model assessment for insulin resistance, FPG = fasting plasma glucose; FPI = fasting plasma insulin; CRP = C reactive protein; TGs = triglycerides; TC = total cholesterol; LDL = low-density lipoprotein cholesterol; HDL = high-density lipoprotein cholesterol; SBP = systolic blood pressure; MAP = mean arterial pressure; DBP = diastolic blood pressure; FMD = flow-mediated dilation; cAIx = central augmentation index; cfPWV = carotid–femoral pulse wave velocity; crPWV = carotid–radial pulse wave velocity; cPP = central pulse pressure.

c transformed values used for χ^2^ or ANOVA

a χ^2^ or ANOVA group differences.

Annotations: Lower FMD in the PWH COVID+ group, although not significant, aligns with trends observed in endothelial dysfunction post-COVID-19. Higher cAIx and cfPWV suggest potential arterial stiffness in PWH COVID+ participants, requiring further longitudinal assessment.

## Data Availability

The data for this study include potentially sensitive information related to infectious diseases; thus, extra caution must be taken to ensure individuals who access these data have appropriate ethical approval and data security standards in place. As such, the data are available for study upon request to Principal Investigator Dr. Claudia Martinez (cmartinez5@med.miami.edu).

## References

[1] Jones DL; Salazar AS; Rodriguez VJ; Balise RR; Uribe CS; Morgan K Severe Acute Respiratory Syndrome Coronavirus 2: Vaccine Hesitancy Among Underrepresented Racial and Ethnic Groups with HIV in Miami, Florida. Open Forum Infect. Dis. 2021, 8, ofab154. 34621912 10.1093/ofid/ofab154 PMC8083672

[2] Clerkin KJ; Fried JA; Raikhelkar J; Sayer G; Griffin JM; Masoumi A; Jain SS; Burkhoff D; Kumaraiah D; Rabbani L; COVID-19 and Cardiovascular Disease. Circulation 2020, 141, 1648–1655. 32200663 10.1161/CIRCULATIONAHA.120.046941

[3] Holland DJ; Blazak PL; Martin J; Broom J; Poulter RS; Stanton T Myocarditis and Cardiac Complications Associated with COVID-19 and mRNA Vaccination: A Pragmatic Narrative Review to Guide Clinical Practice. Heart Lung Circ. 2022, 31, 924–933. 35398005 10.1016/j.hlc.2022.03.003 PMC8984702

[4] Tolu-Akinnawo O; Adusei Poku F; Elimihele T; League M; Adkins CF; Okafor H Acute Cardiovascular Complications of COVID-19: A Systematic Review. Cureus 2023, 15, e38576. 37168413 10.7759/cureus.38576 PMC10166388

[5] Oliveira LCG; Cruz NAN; Ricelli B; Tedesco-Silva H Jr.; Medina-Pestana JO; Casarini DE Interactions amongst inflammation, renin-angiotensin-aldosterone and kallikrein-kinin systems: Suggestive approaches for COVID-19 therapy. J. Venom. Anim. Toxins Incl. Trop. Dis. 2021, 27, e20200181. 34925477 10.1590/1678-9199-JVATITD-2020-0181 PMC8651214

[6] Zanza C; Romenskaya T; Manetti AC; Franceschi F; La Russa R; Bertozzi G; Maiese A; Savioli G; Volonnino G; Longhitano Y; Cytokine Storm in COVID-19: Immunopathogenesis and Therapy. Medicina 2022, 58, 144. 35208467 10.3390/medicina58020144 PMC8876409

[7] Ambrosino P; Calcaterra IL; Mosella M; Formisano R; D’Anna SE; Bachetti T; Marcuccio G; Galloway B; Mancini FP; Papa A; Endothelial Dysfunction in COVID-19: A Unifying Mechanism and a Potential Therapeutic Target. Biomedicines 2022, 10, 812. 35453563 10.3390/biomedicines10040812 PMC9029464

[8] Webel AR; Schexnayder J; Cioe PA; Zuñiga JA A Review of Chronic Comorbidities in Adults Living With HIV: State of the Science. J. Assoc. Nurses AIDS Care 2021, 32, 322–346. 33595986 10.1097/JNC.0000000000000240 PMC8815414

[9] Schexnayder J; Longenecker CT; Muiruri C; Bosworth HB; Gebhardt D; Gonzales SE; Hanson JE; Hileman CO; Okeke NL; Sico IP; Understanding constraints on integrated care for people with HIV and multimorbid cardiovascular conditions: An application of the Theoretical Domains Framework. Implement. Sci. Commun. 2021, 2, 17. 33579396 10.1186/s43058-021-00114-z PMC7881687

[10] Finlayson-Trick E; Tam C; Wang L; Dawydiuk N; Salters K; Trigg J; Pakhomova T; Marante A; Sereda P; Wesseling T; Access to care and impact on HIV treatment interruptions during the COVID-19 pandemic among people living with HIV in British Columbia. HIV Med. 2024, 25, 1007–1018. 38720646 10.1111/hiv.13654

[11] Baumer Y; Farmer N; Premeaux TA; Wallen GR; Powell-Wiley TM Health Disparities in COVID-19: Addressing the Role of Social Determinants of Health in Immune System Dysfunction to Turn the Tide. Front. Public Health 2020, 8, 559312. 33134238 10.3389/fpubh.2020.559312 PMC7578341

[12] Rolls S; Denneny E; Marcus R; O’Connell R Tackling cardiovascular co-morbidities in HIV-positive patients: Who, how and where? J. Int. AIDS Soc. 2014, 17 (Suppl. S3), 19725. 25397471 10.7448/IAS.17.4.19725 PMC4225426

[13] Collins LF Persons With Human Immunodeficiency Virus and the Coronavirus Disease 2019 Pandemic: A Viral Synergy of Biology and Sociology. Clin. Infect. Dis. 2021, 73, e2106–e2108. 33161433 10.1093/cid/ciaa1715 PMC7717215

[14] Brown LB; Spinelli MA; Gandhi M The interplay between HIV and COVID-19: Summary of the data and responses to date. Curr. Opin. HIV AIDS. 2021, 16, 63–73. 33186229 10.1097/COH.0000000000000659 PMC7735216

[15] Nejadghaderi SA; Heidari A; Shakerian N; Saghazadeh A; Rezaei N Cardiovascular system is at higher risk of affecting by COVID-19. Acta Biomed. 2020, 91, e2020018. 10.23750/abm.v91i3.9718 PMC7717008 32921715

[16] Pelisek J; Reutersberg B; Greber UF; Zimmermann A Vascular dysfunction in COVID-19 patients: Update on SARS-CoV-2 infection of endothelial cells and the role of long non-coding RNAs. Clin. Sci. 2022, 136, 1571–1590. 10.1042/CS20220235 PMC9652506 36367091

[17] Jones DL; Morgan KE; Martinez PC; Rodriguez VJ; Vazquez A; Raccamarich PD; Alcaide ML COVID-19 Burden and Risk Among People With HIV. J. Acquir. Immune Defic. Syndr. 2021, 87, 869–874. 33999015 10.1097/QAI.0000000000002656 PMC8136457

[18] Pournamdari AB; Hsue PY; Parikh RV HIV-Associated Cardiovascular Disease: Beyond the Macrovascular. J. Am. Heart Assoc. 2023, 12, e031876. 37947107 10.1161/JAHA.123.031876 PMC10727305

[19] Ma Z; Yang KY; Huang Y; Lui KO Endothelial contribution to COVID-19: An update on mechanisms and therapeutic implications. J. Mol. Cell. Cardiol. 2022, 164, 69–82. 34838588 10.1016/j.yjmcc.2021.11.010 PMC8610843

[20] Veluswamy P; Wacker M; Stavridis D; Reichel T; Schmidt H; Scherner M; Wippermann J; Michels G The SARS-CoV-2/Receptor Axis in Heart and Blood Vessels: A Crisp Update on COVID-19 Disease with Cardiovascular Complications. Viruses 2021, 13, 1346. 34372552 10.3390/v13071346 PMC8310117

[21] Giordo R; Paliogiannis P; Mangoni AA; Pintus G SARS-CoV-2 and endothelial cell interaction in COVID-19: Molecular perspectives. Vasc. Biol. 2021, 3, R15–R23. 33659858 10.1530/VB-20-0017 PMC7923034

[22] Cai CW; Sereti I Residual immune dysfunction under antiretroviral therapy. Semin Immunol. 2021, 51, 101471. 33674177 10.1016/j.smim.2021.101471 PMC8410879

[23] Dirajlal-Fargo S; Funderburg N HIV and cardiovascular disease: The role of inflammation. Curr. Opin. HIV AIDS 2022, 17, 286–292. 35938462 10.1097/COH.0000000000000755 PMC9370832

[24] Surial B; Chammartin F; Damas J; Calmy A; Haerry D; Stöckle M; Schmid P; Bernasconi E; Fux CA; Tarr PE; Impact of Integrase Inhibitors on Cardiovascular Disease Events in People with Human Immunodeficiency Virus Starting Antiretroviral Therapy. Clin. Infect. Dis. 2023, 77, 729–737. 37157869 10.1093/cid/ciad286 PMC10495132

[25] Parra-Rodriguez L; Sahrmann JM; Butler AM; Olsen MA; Powderly WG; O’Halloran JA Antiretroviral Therapy and Cardiovascular Risk in People with HIV in the United States-An Updated Analysis. Open Forum Infect Dis. 2024, 11, ofae485. 39296337 10.1093/ofid/ofae485 PMC11409880

[26] Lv T; Cao W; Li T HIV-Related Immune Activation and Inflammation: Current Understanding and Strategies. J. Immunol. Res. 2021, 2021, 7316456. 34631899 10.1155/2021/7316456 PMC8494587

[27] Hwa SH; Snyman J; Bernstein M; Ganga Y; Cele S; Muema D; Tan CW; Khan K; Karim F; Hanekom W; Association Between Human Immunodeficiency Virus Viremia and Compromised Neutralization of Severe Acute Respiratory Syndrome Coronavirus 2 Beta Variant. J. Infect. Dis. 2023, 227, 211–220. 35975942 10.1093/infdis/jiac343 PMC9452105

[28] Ssentongo P; Heilbrunn ES; Ssentongo AE; Advani S; Chinchilli VM; Nunez JJ; Du P Epidemiology and outcomes of COVID-19 in HIV-infected individuals: A systematic review and meta-analysis. Sci. Rep. 2021, 11, 6283. 33737527 10.1038/s41598-021-85359-3 PMC7973415

[29] Bhaskaran K; Rentsch CT; MacKenna B; Schultze A; Mehrkar A; Bates CJ; Eggo RM; Morton CE; Bacon SC; Inglesby P; HIV infection and COVID-19 death: A population-based cohort analysis of UK primary care data and linked national death registrations within the OpenSAFELY platform. Lancet HIV 2021, 8, e24–e32. 33316211 10.1016/S2352-3018(20)30305-2 PMC7773630

[30] Western Cape Department of Health; National Institute for Communicable Diseases, South Africa. Risk factors for coronavirus disease 2019 (COVID-19) death in a population cohort study from the Western Cape Province, South Africa. Clin. Infect. Dis. 2021, 73, e2005–e2015. 32860699 10.1093/cid/ciaa1198 PMC7499501

[31] Del Amo J; Polo R; Moreno S; Jarrín I; Hernán MA SARS-CoV-2 infection and coronavirus disease 2019 severity in persons with HIV on antiretroviral treatment. AIDS 2022, 36, 161–168. 34934017 10.1097/QAD.0000000000003132 PMC8824311

[32] Augello M; Bono V; Rovito R; Tincati C; Marchetti G Immunologic Interplay Between HIV/AIDS and COVID-19: Adding Fuel to the Flames? Curr. HIV/AIDS Rep. 2023, 20, 51–75. 36680700 10.1007/s11904-023-00647-z PMC9860243

[33] Matsuzawa Y; Kwon TG; Lennon RJ; Lerman LO; Lerman A Prognostic value of flow‚ Äêmediated vasodilation in brachial artery and fingertip artery for cardiovascular events: A systematic review and meta-analysi. J. Am. Heart Assoc. 2015, 4, e002270. 26567372 10.1161/JAHA.115.002270 PMC4845238

[34] Alcaide ML; Nogueira NF; Salazar AS; Montgomerie EK; Rodriguez VJ; Raccamarich PD; Barreto IT; McGaugh A; Sharkey ME; Mantero AM; A Longitudinal Analysis of SARS-CoV-2 Antibody Responses Among People with HIV. Front. Med. 2022, 9, 768138. 10.3389/fmed.2022.768138 PMC8940197 35330585

[35] Martinez CA; Rikhi R; Pester MS; Parker M; Gonzalez A; Larson M; Chavez J; Mendez A; Raines JK; Kolber MA; Abacavir antiretroviral therapy and indices of subclinical vascular disease in persons with HIV. PLoS ONE 2022, 17, e0264445. 35271614 10.1371/journal.pone.0264445 PMC8912137

[36] Harris PA; Taylor R; Thielke R; Payne J; Gonzalez N; Conde JG Research electronic data capture (REDCap)—A metadata-driven methodology and workflow process for providing translational research informatics support. J. Biomed. Inform. 2009, 42, 377–381. 18929686 10.1016/j.jbi.2008.08.010 PMC2700030

[37] Matthews DR; Hosker JP; Rudenski AS; Naylor BA; Treacher DF; Turner RC Homeostasis model assessment: Insulin resistance and beta-cell function from fasting plasma glucose and insulin concentrations in man. Diabetologia 1985, 28, 412–419. 3899825 10.1007/BF00280883

[38] Friedewald WT; Levy RI; Fredrickson DS Estimation of the concentration of low-density lipoprotein cholesterol in plasma, without use of the preparative ultracentrifuge. Clin. Chem. 1972, 18, 499–502. 4337382

[39] Feinstein MJ; Hsue PY; Benjamin LA; Bloomfield GS; Currier JS; Freiberg MS; Grinspoon SK; Levin J; Longenecker CT; Post WS; Characteristics, prevention, and management of cardiovascular disease in people living with HIV: A scientific statement from the American Heart Association. Circulation 2019, 140, e98–e124. 31154814 10.1161/CIR.0000000000000695 PMC7993364

[40] Jud P; Gressenberger P; Muster V; Avian A; Meinitzer A; Strohmaier H; Sourij H; Raggam RB; Stradner MH; Demel U; Evaluation of Endothelial Dysfunction and Inflammatory Vasculopathy After SARS-CoV-2 Infection-A Cross-Sectional Study. Front. Cardiovasc. Med. 2021, 8, 750887. 34722682 10.3389/fcvm.2021.750887 PMC8549830

[41] Otifi HM; Adiga BK Endothelial Dysfunction in COVID-19 Infection. Am. J. Med. Sci. 2022, 363, 281–287. 35093394 10.1016/j.amjms.2021.12.010 PMC8802031

[42] Bianconi V; Mannarino MR; Figorilli F; Schiaroli E; Cosentini E; Batori G; Marini E; Sahebkar A; Grignani F; Gidari A; Low brachial artery flow-mediated dilation predicts worse prognosis in hospitalized patients with COVID-19. J. Clin. Med. 2021, 10, 5456. 34830738 10.3390/jcm10225456 PMC8621380

[43] Peng J; Guo W; Li P; Leng L; Gao D; Yu Z; Huang J; Guo J; Wang S; Hu M; Long-term effects of COVID-19 on endothelial function, arterial stiffness, and blood pressure in college students: A pre-post-controlled study. BMC Infect. Dis. 2024, 24, 742. 39068389 10.1186/s12879-024-09646-w PMC11282677

[44] Aljadah M; Khan N; Beyer AM; Chen Y; Blanker A; Widlansky ME Clinical Implications of COVID-19-Related Endothelial Dysfunction. JACC Adv. 2024, 3, 101070. 39055276 10.1016/j.jacadv.2024.101070 PMC11269277

[45] Sánchez-Santillán RN; Sierra-Vargas MP; González-Islas D; Aztatzi-Aguilar OG; Pérez-Padilla R; Orea-Tejeda A; Debray-García Y; Ortega-Romero M; Keirns-Davis C; Loaeza-Roman A; Endothelial biomarkers (Von willebrand factor, BDCA3, urokinase) as predictors of mortality in COVID-19 patients: Cohort study. BMC Pulm. Med. 2024, 24, 325. 38965511 10.1186/s12890-024-03136-0 PMC11229487

[46] Rombini MF; Cecchini D; Diana Menendez S; Calanni L; Cuini R; Obieta E; Greco MM; Morales F; Morganti L; Migazzi C; Tenofovir-Containing Antiretroviral Therapy and Clinical Outcomes of SARS-CoV-2 Infection in People Living with HIV. Viruses 2023, 15, 1127. 37243213 10.3390/v15051127 PMC10223653

